# Navigating the new frontier: technical, humanistic, and ethical skills in AI-augmented stroke care for vascular neurologists: a scoping review

**DOI:** 10.31744/einstein_journal/2026RW2050

**Published:** 2026-09-15

**Authors:** João Brainer Clares de Andrade, Rafael Pádua Gomes, Alexandre Cristiuma Robles, George Nunes Mendes, Thales Pardini Fagundes, Thiago Santos Carneiro

**Affiliations:** 1 Universidade Federal de São Paulo São Paulo SP Brazil Universidade Federal de São Paulo, São Paulo, SP, Brazil.; 2 Hospital Israelita Albert Einstein São Paulo SP Brazil Hospital Israelita Albert Einstein. São Paulo, SP, Brazil.; 3 Laboratório de Bioengenharia Instituto de Tecnologia Aeronáutica São José dos Campos SP Brazil Laboratório de Bioengenharia, Instituto de Tecnologia Aeronáutica, São José dos Campos, SP, Brazil.; 4 Faculdade de Medicina Centro Universitário São Camilo São Paulo SP Brazil Faculdade de Medicina, Centro Universitário São Camilo, São Paulo, SP, Brazil.; 5 Centre Hospitalier I’Universite de Montreal Quebec Canada Centre Hospitalier de I’Universite de Montreal, Quebec, Canada.; 6 Hospital de Amor Barretos SP Brazil Hospital de Amor, Barretos, SP, Brazil.; 7 Department of Neurocritical Care University of Florida Gainesville USA Department of Neurocritical Care, University of Florida, Gainesville, USA.

**Keywords:** Vascular neurology, Artificial Intelligence, Humanities, Bioethics, Ethics, Stroke, Decision support systems, clinical

## Abstract

**Background:**

Artificial Intelligence is increasingly embedded in stroke care, yet clear guidance on the competencies required for its safe and ethical use in vascular neurology is lacking.

**Objective:**

To map the literature on Artificial Intelligence applications across the stroke care continuum and to synthesize the technical, ethical-legal, and humanistic competencies needed for Artificial Intelligence-augmented clinical practice.

**Data sources:**

PubMed/MEDLINE, Embase, Scopus, Google Scholar, and targeted gray literature from professional and regulatory bodies.

**Eligibility criteria:**

Peer-reviewed studies, reviews, and consensus statements or guidance documents addressing Artificial Intelligence in stroke care or related ethicallegal and humanistic competencies. Case reports, exclusively preclinical studies, and non-scholarly publications were excluded.

**Methods:**

A scoping review was conducted according to PRISMA-ScR and JBI guidance, with independent screening and narrative thematic synthesis.

**Results:**

From 1,426 records, 74 studies were included. Validated Artificial Intelligence applications were identified in hyperacute imaging (e.g., automated ASPECTS and large-vessel occlusion detection), prognostication, complication prediction, and rehabilitation monitoring, with improvements in speed and precision. Key gaps included limited external validation, insufficient bias assessment, and weak implementation governance. Three competency domains emerged: technical (data literacy and model evaluation), ethical-legal (accountability, privacy and equity), and humanistic (empathy, communicationand shared decision-making). We propose “clinical-Artificial Intelligence correlation” as a core competency integrating algorithmic outputs with clinical judgment.

**Conclusion:**

Artificial Intelligence can enhance stroke care when combined with rigorous clinical oversight and strong ethical and humanistic foundations.

**Registration:**

10.17605/OSF.IO/G4W9J

## INTRODUCTION

### The evolving paradigm of vascular neurology

Neurology is in the midst of a profound transformation, a “neurology revolution” driven by the confluence of rapid advancements in therapeutics and the exponential growth of Artificial Intelligence (AI).^(1)^ For vascular neurology, a specialty defined by its time-critical decision-making and high-stakes outcomes, this revolution is not a distant prospect but a present-day reality.^(2)^Artificial Intelligence-powered algorithms are moving from research laboratories into the emergency department, the neuro-ICU, and the outpatient clinic, fundamentally altering the diagnostic and therapeutic landscape of stroke care.^(2-4)^ This technological surge presents an urgent and defining challenge for the modern neurologist, particularly neurologists at the start of their careers: how to harmoniously integrate the immense analytical power of AI with the foundational, irreplaceable human aspects of medicine.^(1,5,6)^ Nevertheless, a gap remains in the literature: there are no clear guidelines on how to integrate these AI tools into vascular neurology practice without losing sight of the humanistic and ethical foundations of care.

This challenge brings into sharp focus a central tension within contemporary healthcare.” As argued in the seminal work Deep Medicine^(7)^the practice of medicine has, in many ways, become increasingly “inhuman”. Clinicians are often overwhelmed by administrative duties, particularly the clerical burden of electronic health records (EHRs), leading to a well-documented erosion of the doctor-patient relationship—the very heart of medicine.^(8,9)^In this context, AI emerges as a technology with dual and divergent potential. On one hand, it could exacerbate this dehumanization, reducing patients to data points and clinical practice to algorithmic protocols. On the one hand, if implemented with intention and wisdom, AI offers the tantalizing prospect of restoring human connection.^(5,6)^ Artificial Intelligence can free up time for what truly matters: attentive listening,^(10)^thorough examination, empathy, and for building the trust that underpins all healing.^(6,7)^

This scoping review aims to map the literature on Artificial Intelligence applications across the stroke care continuum and to synthesize the technical, ethical-legal, and humanistic competencies required for AI-augmented vascular neurology practice, in order to identify current gaps and inform future research and training.

## METHODS

We conducted a scoping review to systematically map the breadth and nature of the evidence at the intersection of vascular neurology, artificial intelligence, ethics, and humanism, as well as to clarify key concepts, identify gaps, and inform future research and education. A scoping approach was chosen over a systematic review because the field encompasses heterogeneous study designs, outcomes, and conceptual papers for which quantitative synthesis is neither feasible nor appropriate at this time. Reporting followed the PRISMA-ScR guideline,^(11)^ and methodological decisions were aligned with JBI guidance,^(12)^ and the refinement proposed Levac et al.^(13)^ to the framework developed by Arksey et al.^(14)^

The ethical and regulatory analysis was grounded in official guidelines and position statements from key national and international professional and regulatory bodies, including the American Medical Association (AMA),^(15,16)^ the World Medical Association (WMA),^(17)^ the Committee on Publication Ethics (COPE), and the International Committee of Medical Journal Editors (ICMJE).

### Inclusion criteria

We included peer-reviewed original studies, reviews, consensus statements and guidance documents, and select methodological papers addressing AI applications relevant to stroke care (including risk stratification, hyperacute diagnosis, inpatient management, rehabilitation and secondary prevention) or the ethical-legal and humanistic competencies required for AI-augmented practice.

### Exclusion criteria

We excluded isolated case reports, purely preclinical or simulation-only studies with no clinical linkage, non-scholarly commentaries, and unretrievableitems. Searches covered the period from January 1, 2020, through June 30, 2025 with the final search conducted on June 30, 2025. We limited inclusion to English-language records to enable consistent screening and synthesis across reviewers and to reflect most technical and regulatory sources in this domain.

The database search yielded 1,426 records. After the removal of 612 duplicates and 38 records for other reasons, 892 records underwent title and abstract screening. Of these, 604 were excluded. The Full texts were sought n of 288 reports were sought, of which 21 could not be retrieved. A total of 267 full-text articles were assessed for eligibility, resulting in 74 studies being included in the final scoping review (Figure 1).

**Figure 1:**
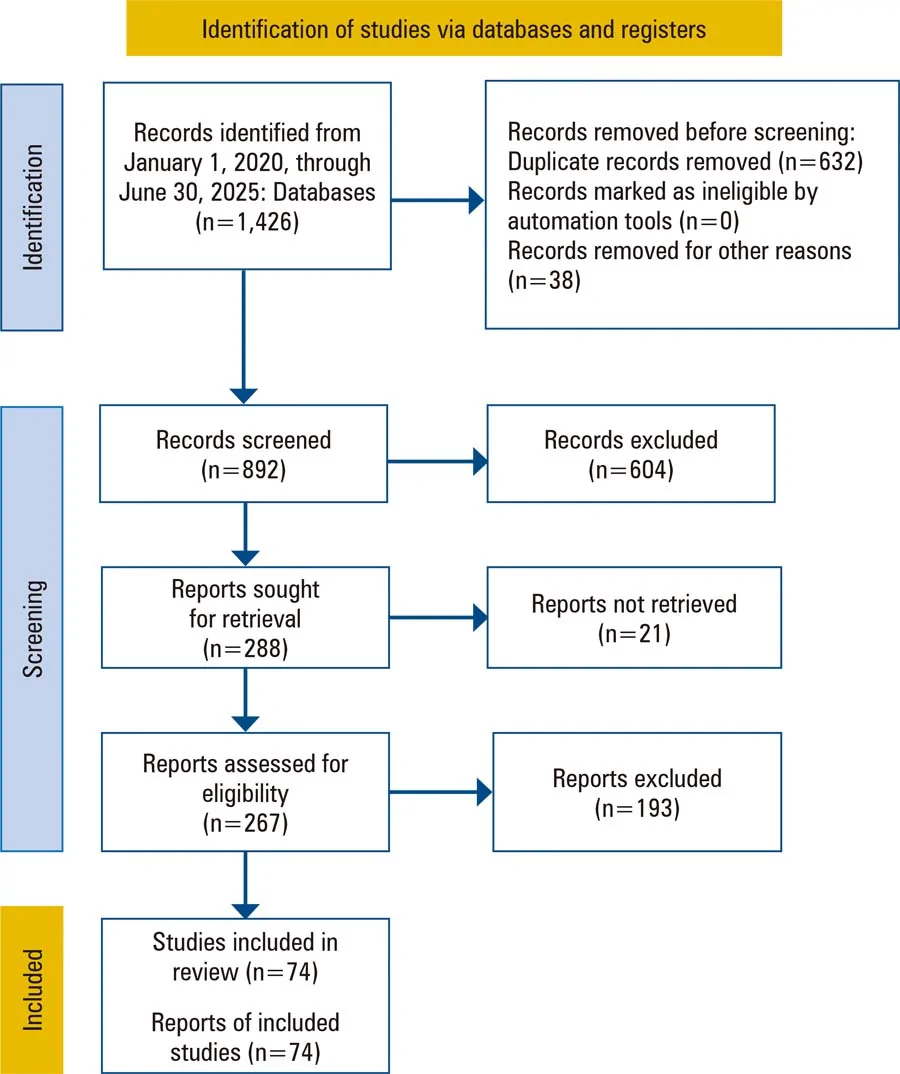
Studies selection process

### Search strategy

We searched PubMed/MEDLINE, Embase, and Scopus, and screened Google Scholar for forward/backward citation chasing. Targeted gray literature included policy and position statements from major bodies (e.g., AMA, WMA, COPE and ICMJE) and regulatory guidance relevant to AI in healthcare. A medical librarian reviewed the search strategy. The full search strategy is provided in Table 1S, Supplementary Material.

Records were deduplicated and screened in two stages (title and abstract screening, followed by full text screening) by two independent reviewers using prespecified criteria. Disagreements were resolved by consensus or adjudication by a third reviewer.

We piloted a standardized data-charting form on a sample of studies and refined it iteratively data charting was performed in duplicate. Extracted items included: bibliographic data; study aim, design, context; patient population; stroke phase addressed; AI task (e.g., ASPECTS automation, LVO detection, and outcome prediction); input data and dataset characteristics (size, source and geography); performance metrics (AUC, sensitivity,specificity and calibration); external validation status; regulatory status, if applicable; comparators; key findings; fairness and bias assessment; and implementation outcomes (e.g) (effects on time to treatment).

Given the expected heterogeneity in concepts, study designs, and outcomes, we conducted a narrative, thematic synthesis mapping the evidence across the stroke-care continuum (hyperacute, inpatient, rehabilitation and secondary prevention) and competency domains (technical, ethical-legal, humanistic). No quantitative synthesis (meta-analysis) was conducted

Consistent with the objectives of a scoping review, no formal risk of bias or methodological quality assessment of individual studies was conducted. The purpose of this review was to map the breadth and nature of the evidence rather than to appraise or pool study outcomes.

Following data charting, studies were grouped thematically according to (a) the stage of the stroke care continuum, (b) the type of AI application, and (c) the competency domain addressed (technical, ethical-legal, or humanistic). Findings are presented through descriptive summaries, tables, and conceptual frameworks without quantitative aggregation.

### The modern stroke neurologist’s toolkit: a synthesis of technical expertise and AI augmentation

The contemporary vascular neurologist is equipped with a rapidly expanding toolkit in which AI is no longer a futuristic concept but a practical, integrated component.^(4,8,18)^ These technologies are enhancing precision and speed across the entire stroke care continuum, from the initial moments of diagnosis to long-term recovery. For the early-career physician, mastering this AI-augmented toolkit is becoming a core competency.^(19-21)^

In the hyperacute phase of ischemic stroke, where “time is brain,” AI is making its most dramatic impact by accelerating and refining diagnosis. The ability of machine learning algorithms to process vast amounts of imaging data with remarkable speed and accuracy is revolutionizing the front lines of stroke care. One important application is automated image interpretation. Neuroimaging remains the cornerstone of stroke diagnosis, and AI has demonstrated expert-level performance in image interpretation.^(22)^ For the early-career neurologist on call, this translates into powerful decision support. Artificial Intelligence-driven software can automatically calculate the Alberta Stroke Program Early CT Score (ASPECTS) from noncontrast head CTs scans, providing a rapid and standardized assessment of early ischemic changes.^(22,23)^ Similarly, AI-powered classifiers can screen CT angiography images (CTAs) to detect large vessel occlusions (LVOs) with high sensitivity, flagging potential candidates for mechanical thrombectomy moments after the scan is completed.^(24)^ Deep learning models are also being deployed to automatically segment and quantify infarctcore in diffusion-weighted imaging (DWI), offering a more objective measure of ischemic injury than visual estimation alone.^(26)^ For example, in a multicenter telestroke network,^(27)^ implementation of an AI platform for LVO detection and care coordination was associated with a mean reduction of approximately 40 minutes in the time from patient arrival to neurointerventionalist contact, compared with a non-AI workflow. This time gain illustrates the practical impact of AI on expediting thrombectomy pathways and may translate into improved clinical outcomes. These tools do not replace the neurologist or radiologist but act as crucial second readers, reducing cognitive load and significantly shortening the time to critical treatment decisions.

### Precision in treatment and prognostication

The traditional approach to stroke care has relied on treatment protocols applied broadly across patient populations. Artificial Intelligence is a key driver of the shift toward precision medicine, tailoring interventions and predictions to the unique characteristics of each individual patient.

One of the most powerful applications of AI is prognostication.^(28)^ By integrating vast datasets – including clinical information from EHR, demographic data, and imaging features – machine learning models can predict functional outcomes after stroke with significantly greater accuracy than traditional scoring systems.^(28)^ For the neurologist, this provides an evidence-based tool for counseling patients and their families, setting realistic expectations for recovery, and guiding decisions about the intensity and focus of rehabilitation.

Artificial Intelligence is beginning to help answer one of the most difficult questions in medicine: which treatment is best for this specific patient? Instead of relying solely on population-level data from clinical trials, AI models can estimate the “conditional average treatment effect” (CATE).^(29)^ This technique analyzes an individual patient’s complex data profile to predict the patient’s likely benefit from a specific intervention, such as thrombolysis or thrombectomy. This approach embodies the movement away from treating the hypothetical”average patient” and toward a truly personalized therapeutic strategy, ensuring that interventions are directed toward those most likely to benefit. In practical terms, these techniques may allow clinicians to estimate, for example, whether an older patient with multiple comorbidities is likely to benefit from thrombectomy beyond the standard time window, rather than relying solely on population-level criteria. Thus, the neurologist can personalize the therapeutic strategy—treating the patient, not the statistical average.^(29)^

Some of these applications are presented in table 1.

**Table 1:** Artificial Intelligence applications across the stroke care continuum

Stage of care	AI application	Data modality	Clinical Impact
Prevention & Risk Stratification	Atrial fibrillation detection	ECG/PPG from wearable devices ^(30,31)^	Early identification of a major stroke risk factor, enabling timely initiation of anticoagulation
	Polygenic Risk Score (PRS) prediction	Genomic data, retinal images^(32,33)^	Individualized risk assessment for primary prevention strategies
Hyperacute Diagnosis	Large vessel occlusion (LVO) detection	CT Angiography (CTA)^(24)^	Rapid identification of patients eligible for mechanical thrombectomy, reducing time-to-treatment
	Automated ASPECTS calculation	Non-contrast CT^(23)^	Rapid, standardized assessment of early ischemic changes to guide treatment decisions
	Ischemic core segmentation	CT Perfusion, MR-DWI^(34)^	Objective quantification of infarct volume, improving patient selection for reperfusion therapies
Treatment & Prognostication	Functional outcome prediction	EHR data, imaging features^(26,29,35)^	Personalized prognostication to guide goals-of-care discussions and rehabilitation planning
	Treatment effect estimation (CATE)	Clinical trial data, imaging^(28)^	Tailoring therapeutic decisions such as thrombectomy to the individual patient most likely to benefit
Inpatient Management	Prediction of complications	EHR data, vital signs^(36,37)^	Proactive identification of patients at risk for complications such as sepsis or hemorrhagic transformation
Rehabilitation & Secondary Prevention	Remote motor function monitoring	Wearable accelerometer data^(38,39)^	Continuous, objective tracking of recovery and adherence to therapy outside the clinic
	AI-powered neurorehabilitation	Brain-Computer Interfaces (BCIs)^(40,41)^	Facilitating motor function restoration through neuroprosthetics and targeted neural decoding

AI: Artificial Intelligence; AF: Atrial Fibrillation; ASPECTS: Alberta Stroke Program Early CT Score; BCI: Brain-Computer Interface; CATE: Conditional Average Treatment Effect; CT: Computed Tomography; CTA: CT Angiography; DWI: Diffusion-Weighted Imaging; HER: Electronic Health Record; ECG: Electrocardiogram; LVO: Large Vessel Occlusion; MR: Magnetic Resonance; PPG: Photoplethysmography; PRS: Polygenic Risk Score.

### The ethical and legal gauntlet: ensuring responsible innovation in neurological practice

The rapid integration of AI into neurology brings with it a host of complex ethical and legal challenges that early-career physicians must be prepared to navigate^(42,43)^ The promise of technological advancement must be tempered by a rigorous commitment to patient safety, data privacy, and professional accountability. This requires a deep understanding of the evolving legal frameworks and ethical principles that govern the use of these powerful new tools^(44)^ (Figure 2).

**Figure 2:**
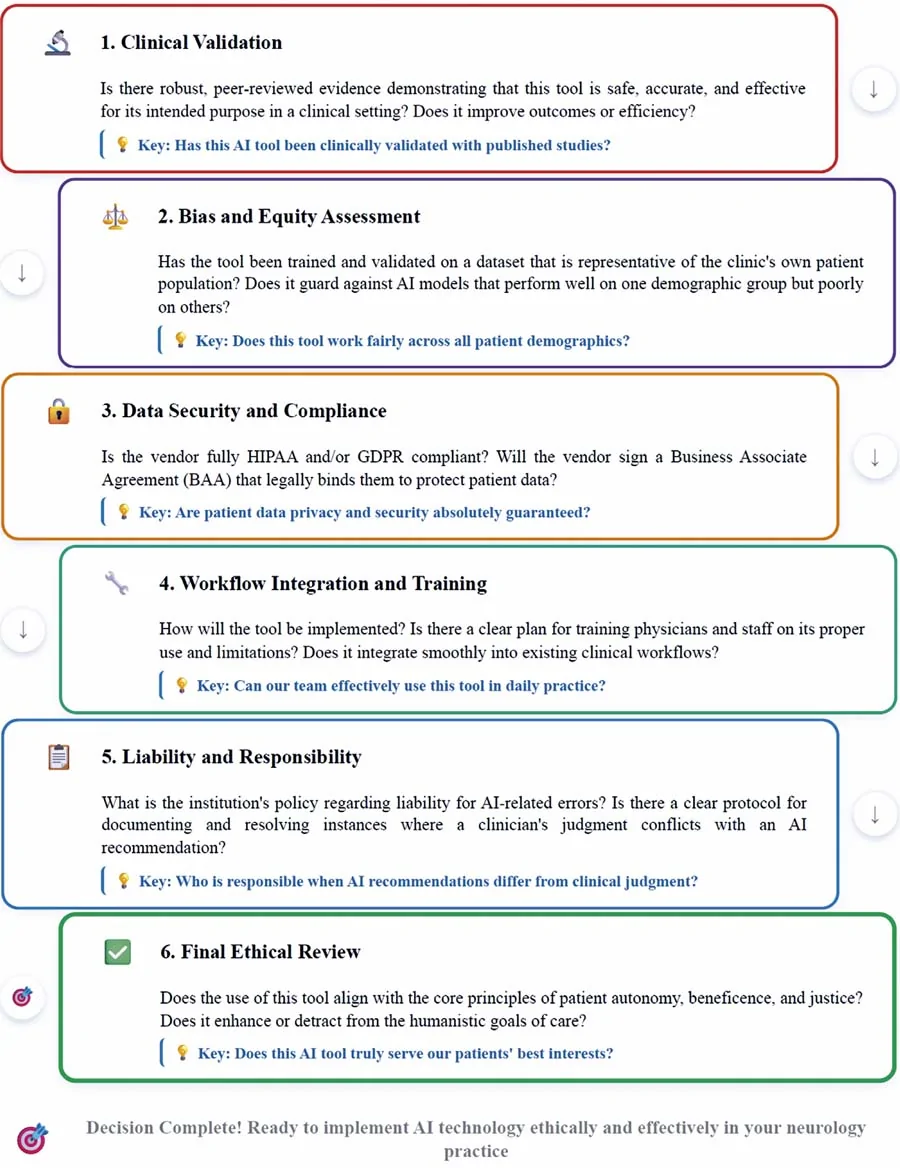
Decision framework for ethical adoption of Artificial Intelligence tools in neurological practice AI: Artificial Intelligence; HIPAA: Health Insurance Portability and Accountability Act; GDPR: General Data Protection Regulation; BAA: Business Associate Agreement; key questions: critical evaluation criteria highlighted for each decision step.

### Accountability and liability in the age of algorithms

Perhaps the most pressing legal question facing the AI-augmented physician is: who is held responsible when an AI tool contributes to patient injury? The traditional legal doctrines of medical malpractice were not designed for a world of “black box” algorithms and shared human-machine decision-making.^(45)^

At the heart of any malpractice claim is the “standard of care” — the level of skill and judgment that a competent physician would exercise in similar circumstances.^(46)^ Artificial Intelligence profoundly complicates this standard, as the legal system has little precedent for AI-assisted malpractice. This creates a difficult paradox for the clinician.^(47)^ A physician could potentially be found negligent for blindly following a flawed AI recommendation that a reasonable physician would have questioned. Conversely, if a physician overrides a correct AI recommendation and the patient experiences an adverse outcome, the physician could also be held liable for failing to appropriately consider the information provided by the technology.^(48)^ This ambiguity places the physician in a precarious legal position.

In the event of an AI-related error, liability is rarely a simple matter. Legal responsibility may be distributed among multiple parties under a shared-responsibility model.^(49,50)^ Potentially responsible parties may include the physician for negligent use, the healthcare institution for failing to provide adequate training or for implementing an unverified or unreliable AI system, and the AI developer for designing a flawed or biased algorithm. Within this framework, the physician often functions as the “learned intermediary”, a legal concept holding that the physician is in the best position to weigh the risks and benefits of a medical product, in this case, an AI tool, for a specific patient.^(48-50)^ This role becomes substantially more challenging when the AI operates as an opaque “black box”,^(45)^ making it difficult for the physician to fully understand or explain how the system arrived at its conclusion.

The profound legal uncertainty surrounding AI-related harm creates a powerful incentive for physicians to practice defensively.^(48,49)^ Without clear legal precedents or established standards of care for AI use, the path of least legal resistance is often to adhere to pre-AI clinical practices and use these powerful new tools merely as secondary, confirmatory aids rather than as primary sources of novel insight.^(49)^ While this conservative approach may be a rational strategy for mitigating an individual physician’s liability risk, it creates a systemic chilling effect that threatens to stifle the very innovation that AI promises. If physicians are discouraged from trusting AI for more than confirming their own initial judgments, the technology’s core potential namely, to detect subtle patterns, uncover nonobvious correlations, and improve diagnostic accuracy beyond the limits of human cognition—may go unrealized.^(47)^ This reality transforms the nature of clinical documentation. The EHR is no longer just a record of care; may also serve as a primary legal document. Every clinical decision to follow or deviate from an AI-generated recommendation should be clearly justified and articulated within the patient’s medical record. The physician’s documented reasoning may provide important evidence supporting the clinical judgment exercised in the event of future claims of negligence.^(42,47,48,50)^ The following example illustrates how this justification could be documented in the medical record:

- AI recommendation: Software X indicated: “Normal cerebral perfusion, no indication for thrombectomy.”
- Clinical assessment: The patient with a significant neurologic deficit and clinical suspicion of a proximal occlusion not visualized on CTA.
- Neurologist’s decision: Proceed with cerebral angiography despite the AI recommendation, because of strong clinical-radiologic correlation indicating an occult occlusion.
- Rationale: The AI model was trained primarily on high-quality images; in this case, motion artifacts may have limited algorithmic detection. The discrepancy and the rationale for further investigation were discussed with the patient and family, and the risks and benefits were considered.

This suggested template demonstrates how to document the AI recommendation, followed by the clinician’s analysis and justified decision. In this way, the medical record reflects the clinical-AI correlation and documents the clinician’s due diligence.

### Data privacy and the ‘sanctity’ of the patient record

The fuel for all medical AI is data, including vast quantities of sensitive patient information. Protecting these data is not merely a technical requirement but a core ethical obligation.^(51)^ Neurologists must be familiar with the regulations that govern this area.

Key principles relevant to AI include the “minimum necessary” standard, which dictates that AI models should only access the data essential for their specific function, and, the requirement for healthcare organizations to have a formal Business Associate Agreement (BAA) with any third-party AI vendor that qualifies as a business associate and handles Protected Health Information (PHI). Patients retain applicable rights to be informed about how and when their data are being used and to accesstheir healt information.^(52)^

A critical and often overlooked issue is the regulatory distinction between different types and sources of data. “Medical” data, such as information contained within an EHR, is subject to established health information privacy requirements, including HIPAA when applicable.^(53)^ However, “health” data, generated by consumer-facing wellness apps, social media platforms, and web searches, exists in a largely unregulated space may fall outside certain traditional health privacy frameworks. Many AI systems designed to analyze social determinants of health (SDOH) may collect, aggregate, or infer information from vast amounts of personal data to create detailed patient profiles without privacy and security protections equivalent to those provided under HIPAA.^(54)^ This practice not only raises significant privacy concerns but also creates a high risk of algorithmic bias, whereby AI models may make discriminatory predictions based on a patient’s post code, purchase history, or online behavior.^(54)^

For example, in Brazil, the protection of health data is governed by the principles of the *Lei Geral de Proteção de Dados Pessoais* (LGPD, Law No. 13,709/2018).^(55)^ Although the LGPD does not explicitly mention “artificial intelligence”, it was inspired by the European GDPR and introduced rights into Brazilian law, such as access to information about automated processing and the right to request review of, automated decisions involving personal data. The LGPD addresses decisions based solely on automated processing and provides data subjects with the right to request a review of such decisions, as established under the applicable legal framework. The LGPD also requires an appropriate legal basis and security measures for the processing of health data. Accordingly, a Brazilian hospital implementing an AI tool must ensure, for example, that there is a lawful basis for processing such as consent or compliance with a legal or regulatory obligation related to healthcare, and that data are anonymized whenever possible and appropriate for the intended purpose. *Lei Geral de Proteção de Dados Pessoais* obligations are enforced by the Brazilian National Data Protection Authority (ANPD), and violations may result in significant administrative sanctions.^(55)^ For example, if a university hospital intends to use patients’ imaging studies to develop a new diagnostic algorithm, it must determine the appropriate legal and ethical basis for the research and implement the required safeguards. Depending on the study design and applicable requirements, these safeguards may include research ethics approval, appropriate consent procedures, transparency measures, access restrictions, and anonymization or pseudonymization of the data.In addition, specific legislation on AI in health is under discussion in the country.Although still under consideration, Bill No. 2,338/2023 establish a Brazilian regulatory framework for AI and indicates that principles such as transparency, nondiscrimination, and accountability will also be central to the national context of AI in medicine.^(55)^ Because the legislative process and the wording of the proposal may change, its status should be updated before publication. These principles reflect the global need to protect privacy in the AI era: whether under HIPAA in the United States, the GDPR in Europe, or the LGPD in Brazil, the use of patient data in algorithms must comply with the requirements applicable to the relevant jurisdiction and ensure appropriate transparency and security, lest public trust be eroded.^(56)^

Several clear ethical boundaries must be observed. First, discussing patient cases with any personally identifiable information on insecure platforms, such as unapproved commercial instant messaging apps, may violate patient privacy and professional ethics.^(57,58)^ Second, the use of patient data, particularly neuroimaging data, for the purpose of training or validating AI algorithms requires an appropriate legal and ethical basis, institutional oversight, and suitable safeguards.^(59,60)^ Depending on the jurisdiction and study context, informed consent may be required or an authorized waiver may apply. Patients should receive appropriate information about the intended use of their data, and identifiable information should be protected through measures such as anonymization or pseudonymization, when appropriate.^(61,62)^

### The ethics of AI-assisted scholarly work

Generative AI tools are rapidly becoming part ofacademic workflows, and their use in scientific writing and publishing is governed by an evolving set of ethical standards.

Leading organizations in the field of publication ethics, including the COPE and the ICMJE, have stated that AI tools cannot be listed as authors of scholarly paper.^(63,64)^ The rationale is that authorship entails responsibility and accountability for the integrity of the work, which an AI system cannot assume.

While AI cannot be credited as an author, its use as a tool in the writing processmay be permissible, provided that applicable journal policies are followed and its use is transparently disclosed when required. Authors who use generative AI to assist with their work—for example, to improve language, grammar, or clarity, should disclose this use in the section specified by the journal. Human authors retain full responsibility for the accuracy, originality, and integrity of themanuscript, including any text drafted or refined using AI.^(63-65)^ Since 2023, major international journals and editorial organizations have adopted or updated policies, emphasizing transparency regarding the use of AI.^(66)^

A significant limitation of using large language models (LLMs) in scientific writing is their propensity for “hallucination” which involves generating plausible-sounding but factually incorrect information, including fabricated or nonexistent citations.^(67)^ Furthermore, because these models are trained on vast text corpora, their outputs may reproduce language that closely resembles existing material, creating a risk of unattributed text reuse

Human authors are responsible for carefully verifying every fact, claim, and reference generated by an AI and for ensuring the scientific and ethical integrity of their work.^(63,64,67)^

### Telemedicine and consultations

Telemedicine has become a standard modality of care in neurology, and its practice is governed by specific legal and ethical requirements designed to uphold the standard of care in a virtual environments.

A fundamental requirement for a telehealth encounter is obtaining and documenting the patient’s informed consent when required by the applicable jurisdiction and type of service.^(68)^ This consent process should include a clear explanation of the nature and potential limitations of a virtual consultation compared with an in-person encounter.

To ensure quality, accountability, continuity of care, telehealth consultation and interprofessional consultations should be appropriately documented in the patient’s medical record. If an encounter is audio- or video-recorded, the recording must be clinically justified, legally authorized, securely stored, and managed in accordance with applicable consent, privacy, confidentiality, and data security requirements. Such recordings may become part of the designated health record and may constitute protected or sensitive health information, depending on the jurisdiction.^(51,69,70)^

The American Medical Association (AMA) and the World Medical Association (WMA) have provided ethical guidance on telemedicine.^(71,72)^ A core principle is that physicians remain responsible for meeting the applicable standard of professional care, regardless of the technology used. Physicians have an ethical duty to ensure that they can conduct a clinically appropriate evaluation, maintain patient privacy and confidentiality, and arrange necessary follow-up care, regardless of whether the encounter is virtual or in person.

In Brazil, resolution No. 2,314/2022 of the Federal Council of Medicine (CFM) regulates telemedicine, and establishes requirements concerning informed consent, medical records, confidentiality, and standards of care. The resolution requires remote care to follow the same ethical standards as in-person care. The LGPD also applies to personal health data processed during teleconsultations in Brazil, requiring appropriate protection of transmitted and stored information. Telestroke networks provide a practical example of this model: AI may support the triage of cases, e.g., by generating app-based alerts for suspected large vessel occlusion, and may assist with clinical documentation.^(4)^

### Deep medicine in practice: Revitalizing the human core of stroke care

While the technical and ethical dimensions of AI are complex, the ultimate goal of its integration into medicine should be simple: to make healthcare more human.^(7,73)^ This requires a conscious effort to leverage technology not as an end in itself but as a means to restore and deepen the physician-patient relationship. This philosophy, central to “*Deep Medicine*”^(7)^ echoes the timeless clinical wisdom of pioneers such as C. Miller Fisher, a prominent figure in the field of stroke neurology.

The most significant humanistic promise of AI is its potential to liberate clinicians from the tasks that have made modern medicine so burdensome.^(7,73)^ A major contributor to physician burnout is the overwhelming burden of clinical documentation and other administrative duties. Artificial Intelligence particularly through ambient scribe technology that can passively capture a patient encounter and generate a draft clinical note, offers a potential solution. By automating documentation and other data-intensive tasks, AI can give physicians the “gift of time”. Studies published in 2024-2025 already demonstrated this benefit: at a tertiary hospital, 74% of physicians reported a reduced documentation burden and lower levels of burnout following the implementation of an AI-assisted clinical documentation system, enabling them to spend more time with patients.^(74)^

This reclaimed time is a highly valuable resource in medicine.^(9)^ The central tenet of deep medicine is that this time must be deliberately reinvested in the patient. It is time that can be spent making eye contact instead of staring at a computer screen. It is time for a more thorough physical examination, more attentive listening, answering questions with presence and patience, and building the rapport and trust that are the true foundations of healing. Evidence suggests that this is not merely an intangible benefit; studies have shown that for every additional minute a clinician spends with a patient, the risk of hospital readmission may decrease. AI, therefore, does not make medicine better by replacing the physician but by freeing the physician to be more fully present with the patient.^(7,9,73)^ (Figure 3).

**Figure 3:**
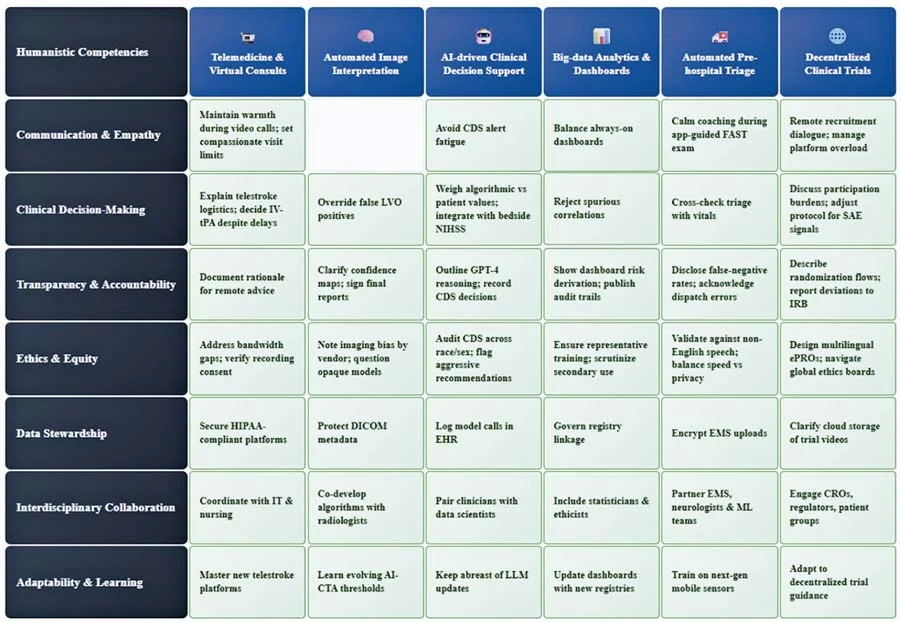
Humanistic competencies in digital stroke care AI: Artificial Intelligence; CDS: Clinical Decision Support; CTA: Computed Tomography Angiography; CTP: CT Perfusion; DCT: Decentralized Clinical Trial; DICOM: Digital Imaging and Communications in Medicine; DWI: Diffusion-Weighted Imaging; EHR: Electronic Health Record; EMS: Emergency Medical Services; ePRO: electronic Patient-Reported Outcome; GDPR: General Data Protection Regulation; HIPAA: Health Insurance Portability and Accountability Act; IRB: Institutional Review Board; IV-tPA: Intravenous Tissue Plasminogen Activator; LLM: Large Language Model; LVO: Large Vessel Occlusion; ML: Machine Learning; NIHSS: National Institutes of Health Stroke Scale; SAE: Serious Adverse Event.

### The “Clinical-AI Correlation” as a new core competency

For generations, a core skill in neurology has been the ability to perform a “clinical-radiological correlation”—synthesizing a patient’s symptoms and signs with findings from neuroimaging. The advent of AI introduces a third, powerful variable into this equation, giving rise to a new, essential competency: the “clinical-AI correlation”.^(75)^ This is a more complex and demanding skill. It requires the neurologist to perform a three-way synthesis, integrating the patient’s clinical presentation, the raw data (such as an MRI images), and the AI’s interpretation of those data.^(76)^

A framework outlining some of the challenges and solutions in this field is presented in figure 4.

**Figure 4:**
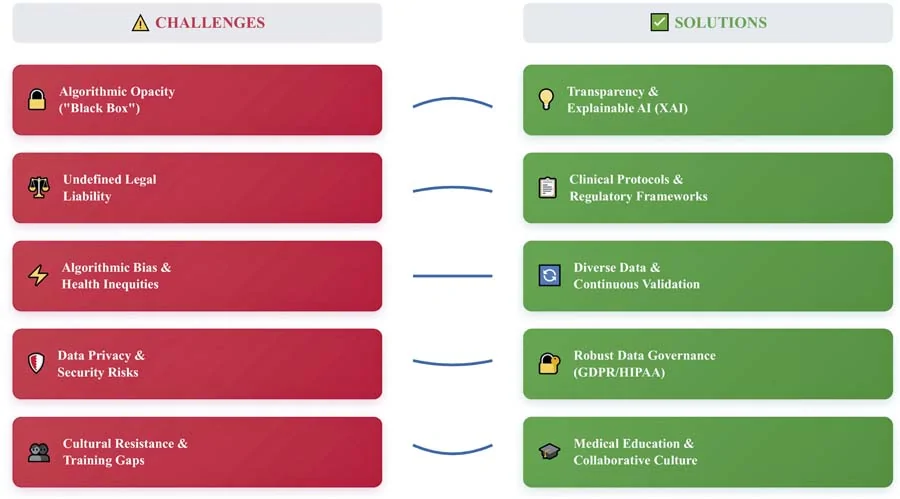
Challenge-solution framework for AI implementation in healthcare settings Framework depicting five key challenges to AI adoption in medicine shown inred paired with corresponding solutions shown in green. Arrows indicate challenge-solution relationships.

GDPR: General Data Protection Regulation; HIPAA: Health Insurance Portability and Accountability Act; XAI: explainable artificial intelligence.

To perform the clinical-AI correlation effectively, the neurologist cannot treat AI as an infallible black box. The neurologist must possess a new layer of knowledge. This includes critical awareness of the data on which the AI system was trained, a recognition of its potential biases such as reduced performance underrepresented demographic groups, and an understanding of its fundamental limitations.^(77-79)^ Therefore, medical education and residency training programs must evolve. It is no longer sufficient to teach early-career neurologists how to use a new AI tool; they must also be taught how to critically evaluate it. This will require incorporating the basic principles of machine learning and data science as well as addressing the profound ethical implications of using potentially flawed or biased datasets in clinical decision-making^(76,78,79)^ (Figure 5).

**Figure 5:**
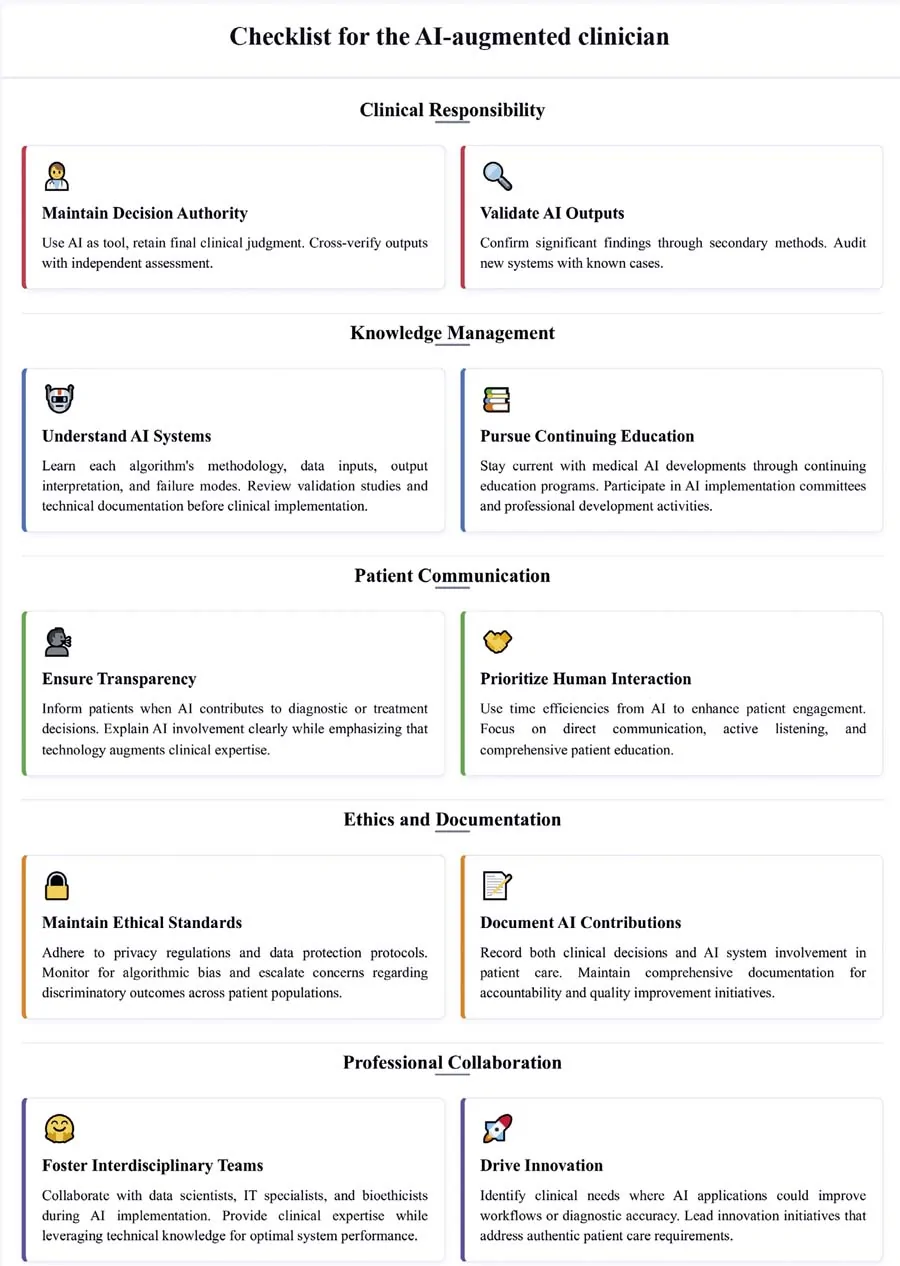
Framework for AI-augmented clinical practice: A comprehensive checklist for healthcare professionals This framework presents ten essential competencies organized into five core domains for clinicians integrating artificial intelligence (AI) into clinical practice. The domains include: (1) clinical responsibility—emphasizing the maintenance of decision- making authority and the validation of AI outputs; (2) knowledge management—focusing on understanding AI systems and pursuing continuing education; (3) patient communication, prioritizing transparency and human interaction; (4) ethics and documentation—maintaining ethical standards and documenting AI contributions; and (5) professional collaboration, fostering interdisciplinary teams and driving innovation. Each competency includes specific, actionable guidelines to ensure the safe, effective, and ethical implementation of AI in healthcare settings.

AI: artificial intelligence; IT: Information Technology.

### Beyond the algorithm: the irreplaceable role of empathy and narrative

Ultimately, the practice of medicine is about caring for human beings, not just interpreting their data. Artificial Intelligence can analyze a CT scan, but it cannot hold a patient’s hand. It can calculate a risk score, but it cannot provide comfort. It can process a million data points, but it cannot understand a single patient’s story, including the patient’s fears, values, and social context, which encompass the social determinants of health that so profoundly shape illness and recovery.^(80-82)^ As Hippocrates famously noted, “It is more important to know what sort of person has a disease than to know what sort of disease a person has”. The physician’s unique and irreplaceable role is to interpreter not only data but also the patient as a whole. ^(82,83)^ Empathy, compassion, and the human touch are not ancillary benefits of care; they are the essence of care itself, qualities that AI can augment but never fully replace.^(7)^

## Conclusion: Forging the neurologist of the future

The incorporation of AI is rapidly reshaping vascular neurology. For early-career neurologists, this should be a moment not of apprehension but of opportunity. Throughout this review, we have shown that excellence in stroke care in the AI era is not a choice between technology and humanism but a mandate to achieve mastery in both. We have addressed a gap in the literature by delineating, in an integrated manner, the technical (e.g., the evaluation and use of imaging algorithms and individualized prognostication), ethical-legal (e.g., navigating issues of liability and privacy under frameworks such as the LGPD), and humanistic (e.g., preserving empathy and the clinical narrative) competencies required for such mastery. We have introduced concepts such as the “clinical-AI correlation”, proposing that critically interpreting algorithmic suggestions alongside patient data should become a new core competency for the modern neurologist. We have also provided practical exemples, from validated tools to checklists, that may serve as a guide for readers in seeking to apply these principles in practice.

The principal insights of this review indicate that, when implemented with critical discernment and purpose, AI can paradoxically make medicine more human: automating the impersonal while freeing physicians to focus on personal interaction. The neurologist of the future will be defined by the capacity work effectively with AI, leveraging its powerto enhance accuracy and efficiency while reaffirming an uncompromising commitment to compassion, ethics, and patient- centered care. In sum, rather than being replaced by AI, the physician who embraces this integration of technology and humanism will remain indispensable to the art and practice of healing in the twenty-first century.

## SUPPLEMENTARY MATERIAL

Supplementary Material
