# Supplementary material for: Navigating the new frontier: technical, humanistic, and ethical skills in AI-augmented stroke care for vascular neurologists: a scoping review
Source: Einstein (Sao Paulo). 2026 Sep 15;24(spe1):eRW2050. doi: 10.31744/einstein_journal/2026RW2050 (PMC13689965; doi:10.31744/einstein_journal/2026RW2050)
Supplement: Supplementary file 1 [file 2317-6385-eins-24-nspe1-eRW2050-suppl01.pdf]

SUPPLEMENTARY MATERIAL

Navigating the new frontier: technical, humanistic, and ethical skills in AI-augmented stroke care for vascular neurologists: a scoping review

João Brainer Clares de Andrade, Rafael Pádua Gomes, Alexandre Cristiuma Robles, George Nunes Mendes, Thales Pardini Fagundes, Thiago Santos Carneiro

DOI: 10.31744/einstein_journal/2026RW2050

Table 1S. Overview of search strategy

Search Period: Inception to August 2025

Databases Searched: PubMed/MEDLINE, Embase, Scopus, Google Scholar

Language Restrictions: No language restrictions applied

Core search concepts

The search strategy was built using three core concept blocks:

Block 1 - Stroke/Cerebrovascular Disease:

- "stroke"
- "cerebrovascular"
- "intracerebral hemorrhage"
- "ischemic stroke"

Block 2 - Artificial Intelligence:

- "artificial intelligence"
- "machine learning"
- "deep learning"

Block 3 - Clinical Applications and Ethical Dimensions:

- "diagnosis"
- "prognosis"
- "triage"
- "clinical decision support"
- "ethics"
- "liability"
- "privacy"
- "data protection"

1. PubMed/MEDLINE

Database Interface: National Library of Medicine (NLM) PubMed

Date of Search: August 2025

Search Strategy:

#1 Search Strategy - Stroke Concepts:

("stroke"[MeSH Terms] OR "stroke"[Title/Abstract] OR "cerebrovascular disorders"[MeSH Terms] OR "cerebrovascular"[Title/Abstract] OR "brain ischemia"[MeSH Terms] OR "ischemic stroke"[Title/Abstract] OR "intracranial hemorrhages"[MeSH Terms] OR "intracerebral hemorrhage"[Title/Abstract] OR "subarachnoid hemorrhage"[MeSH Terms] OR "brain infarction"[MeSH Terms] OR "cerebral infarction"[Title/Abstract])

#2 Search Strategy - Artificial Intelligence Concepts:

("artificial intelligence"[MeSH Terms] OR "artificial intelligence"[Title/Abstract] OR "machine learning"[MeSH Terms] OR "machine learning"[Title/Abstract] OR "deep learning"[MeSH Terms] OR "deep learning"[Title/Abstract] OR "neural networks, computer"[MeSH Terms] OR "neural network"[Title/Abstract] OR "convolutional neural network*"[Title/Abstract] OR "CNN"[Title/Abstract] OR "natural language processing"[MeSH Terms] OR "NLP"[Title/Abstract] OR "computer vision"[Title/Abstract] OR "algorithm*"[Title/Abstract])*

#3 Search Strategy - Clinical Applications:

("diagnosis"[MeSH Terms] OR "diagnosis"[Title/Abstract] OR "prognosis"[MeSH Terms] OR "prognosis"[Title/Abstract] OR "triage"[MeSH Terms] OR "triage"[Title/Abstract] OR "clinical decision support systems"[MeSH Terms] OR "clinical decision support"[Title/Abstract] OR "decision support"[Title/Abstract] OR "image interpretation, computer-assisted"[MeSH Terms] OR "automated image analysis"[Title/Abstract])

continue...

...Continuation

Table 1S. Overview of search strategy

#4 Search Strategy - Ethical and Legal Dimensions:

("ethics, medical"[MeSH Terms] OR "ethics"[Title/Abstract] OR "bioethics"[MeSH Terms] OR "liability, legal"[MeSH Terms] OR "liability"[Title/Abstract] OR "confidentiality"[MeSH Terms] OR "privacy"[Title/Abstract] OR "data protection"[Title/Abstract] OR "GDPR"[Title/Abstract] OR "informed consent"[MeSH Terms] OR "transparency"[Title/Abstract] OR "explainability"[Title/Abstract] OR "interpretability"[Title/Abstract] OR "bias"[Title/Abstract] OR "fairness"[Title/Abstract] OR "equity"[Title/Abstract])

#5 Final Combined Search:

#1 AND #2 AND (#3 OR #4)

Filters Applied:

☐ No date restrictions

☐ No language restrictions

☐ All article types included (original research, reviews, consensus statements, editorials, perspectives)

2. Embase

Database Interface: Elsevier Embase via Ovid

Date of Search: August 2025

Search Strategy:

#1 Stroke Concepts:

'cerebrovascular disease'/exp OR 'stroke'/exp OR 'brain infarction'/exp OR 'brain ischemia'/exp OR 'intracerebral hemorrhage'/exp OR 'subarachnoid hemorrhage'/exp OR stroke:ti,ab OR cerebrovascular:ti,ab OR 'ischemic stroke':ti,ab OR 'ischaemic stroke':ti,ab OR 'intracerebral hemorrhage':ti,ab OR 'intracerebral haemorrhage':ti,ab OR 'cerebral infarction':ti,ab OR 'brain infarction':ti,ab

#2 Artificial Intelligence Concepts:

'artificial intelligence'/exp OR 'machine learning'/exp OR 'deep learning'/exp OR 'neural network'/exp OR 'convolutional neural network'/exp OR 'natural language processing'/exp OR 'computer vision'/exp OR 'artificial intelligence':ti,ab OR 'machine learning':ti,ab OR 'deep learning':ti,ab OR 'neural network':ti,ab OR 'convolutional neural network*':ti,ab OR cnn:ti,ab OR 'natural language processing':ti,ab OR nlp:ti,ab OR 'computer vision':ti,ab OR algorithm*:ti,ab*

#3 Clinical Applications:

'diagnosis'/exp OR 'prognosis'/exp OR 'triage'/exp OR 'clinical decision support system'/exp OR 'computer assisted diagnosis'/exp OR 'image analysis'/exp OR diagnosis:ti,ab OR prognosis:ti,ab OR triage:ti,ab OR 'clinical decision support':ti,ab OR 'decision support':ti,ab OR 'automated image analysis':ti,ab OR 'image interpretation':ti,ab

#4 Ethical and Legal Dimensions:

'medical ethics'/exp OR 'bioethics'/exp OR 'legal liability'/exp OR 'privacy'/exp OR 'data protection'/exp OR 'informed consent'/exp OR ethics:ti,ab OR bioethics:ti,ab OR liability:ti,ab OR privacy:ti,ab OR 'data protection':ti,ab OR gdpr:ti,ab OR 'informed consent':ti,ab OR transparency:ti,ab OR explainability:ti,ab OR interpretability:ti,ab OR bias:ti,ab OR fairness:ti,ab OR equity:ti,ab

#5 Final Combined Search:

#1 AND #2 AND (#3 OR #4)

Limits Applied:

☐ No date restrictions

☐ No language restrictions

☐ All publication types included

☐ Human studies included

☐ Embase records (excluding MEDLINE duplicates for final synthesis)

3. Scopus

Database Interface: Elsevier Scopus

Date of Search: August 2025

Complete Search String:

TITLE-ABS-KEY ((stroke OR cerebrovascular OR "ischemic stroke" OR "ischaemic stroke" OR "intracerebral hemorrhage" OR "intracerebral haemorrhage" OR "subarachnoid hemorrhage" OR "brain infarction" OR "cerebral infarction" OR "brain ischemia"))
AND
TITLE-ABS-KEY (("artificial intelligence" OR "machine learning" OR "deep learning" OR "neural network" OR "convolutional neural network*" OR CNN OR "natural language processing" OR NLP OR "computer vision" OR algorithm*))*
AND
TITLE-ABS-KEY ((diagnosis OR prognosis OR triage OR "clinical decision support" OR "decision support" OR "automated image analysis" OR "image interpretation" OR ethics OR bioethics OR liability OR privacy OR "data protection" OR GDPR OR "informed consent" OR transparency OR explainability OR interpretability OR bias OR fairness OR equity))

Document Type Filters:

☐ Article

☐ Review

☐ Conference Paper

☐ Book Chapter

continue...

...Continuation

Table 1S. Overview of search strategy

☐ Editorial
☐ Letter
☐ Note
☐ Short Survey

Subject Area Filters:

☐ Medicine
☐ Computer Science
☐ Engineering
☐ Health Professions
☐ Decision Sciences
☐ Multidisciplinary

Language: All languages

Date Range: All years

4. Google Scholar

Database Interface: Google Scholar (scholar.google.com)

Date of Search: August 2025

Search Methodology: Forward and backward citation chasing

Primary Search Strategy:

("stroke" OR "cerebrovascular" OR "ischemic stroke" OR "intracerebral hemorrhage") AND ("artificial intelligence" OR "machine learning" OR "deep learning") AND ("diagnosis" OR "prognosis" OR "triage" OR "clinical decision support" OR "ethics" OR "liability" OR "privacy" OR "data protection")

Google Scholar-Specific Approach:

☐ Initial broad search using simplified Boolean operators (Google Scholar has limited Boolean functionality)
☐ Screening of first 200 results sorted by relevance
☐ Forward citation tracking: Using "Cited by" feature for key included studies
☐ Backward citation tracking: Reviewing reference lists of key included studies and relevant review articles
☐ Author-specific searches for key researchers in the field
☐ Search for grey literature including:
  ☐ Technical reports
  ☐ Theses and dissertations
  ☐ Conference proceedings not indexed in traditional databases
  ☐ White papers from technology companies developing AI for stroke care

5. Grey Literature and Additional Sources

5.1 Professional Medical Organizations

American Medical Association (AMA):

Website: www.ama-assn.org

Search Terms: "artificial intelligence", "augmented intelligence", "AI policy", "clinical decision support"

☐ Documents Retrieved:
  ☐ AMA Policy on Augmented Intelligence in Health Care
  ☐ Guidelines for Authors on Use of AI and Language Models
  ☐ Ethical considerations in AI-augmented clinical practice

World Medical Association (WMA):

Website: www.wma.net

Search Terms: "augmented intelligence", "artificial intelligence", "medical care", "ethics"

☐ Documents Retrieved:
  ☐ WMA Statement on Augmented Intelligence in Medical Care

Committee on Publication Ethics (COPE):

Website: publicationethics.org

Search Terms: "artificial intelligence", "authorship", "AI tools", "generative AI"

☐ Documents Retrieved:
  ☐ COPE position statements on AI use in academic publishing
  ☐ Guidelines on disclosure of AI tools in research and writing

continue...

...Continuation

Table 1S. Overview of search strategy

**5.2 Regulatory Bodies**
**U.S. Food and Drug Administration (FDA):**
**Website:** www.fda.gov
**Focus:** AI/ML-based medical devices, clinical decision support, regulatory framework
**Search Terms:** "artificial intelligence", "machine learning", "medical device", "software as medical device", "clinical decision support"
**European Medicines Agency (EMA):**
**Website:** www.ema.europa.eu
**Focus:** EU regulations on AI in healthcare, GDPR compliance
**Search Terms:** "artificial intelligence", "medical device regulation", "data protection", "GDPR"
